# Can successful pregnancy be achieved and predicted from patients with identified *ZP* mutations? A literature review

**DOI:** 10.1186/s12958-022-01046-6

**Published:** 2022-12-08

**Authors:** Juepu Zhou, Meng Wang, Qiyu Yang, Dan Li, Zhou Li, Juan Hu, Lei Jin, Lixia Zhu

**Affiliations:** Reproductive Medicine Center, Tongji Hospital, Tongji Medical College, Huazhong University of Science and Technology, No.1095, Jiefang Road, Wuhan, 430030 China

**Keywords:** *ZP* genes, Mutation, Empty follicle syndrome, Zona pellucida abnormality, Naive Bayes classifier, Prediction model, Pregnancy outcome, Female infertility

## Abstract

**Background:**

In mammals, normal fertilization depends on the structural and functional integrity of the zona pellucida (ZP), which is an extracellular matrix surrounding oocytes. Mutations in *ZP* may affect oogenesis, fertilization and early embryonic development, which may cause female infertility.

**Methods:**

A PubMed literature search using the keywords ‘zona pellucida’, ‘mutation’ and ‘variant’ limited to humans was performed, with the last research on June 30, 2022. The mutation types, clinical phenotypes and pregnancy outcomes were summarized and analyzed. The naive Bayes classifier was used to predict clinical pregnancy outcomes for patients with *ZP* mutations.

**Results:**

A total of 29 publications were included in the final analysis. Sixty-nine mutations of the *ZP* genes were reported in 87 patients with different clinical phenotypes, including empty follicle syndrome (EFS), ZP-free oocytes (ZFO), ZP-thin oocytes (ZTO), degenerated and immature oocytes. The phenotypes of patients were influenced by the types and location of the mutations. The most common effects of *ZP* mutations are protein truncation and dysfunction. Three patients with *ZP1* mutations, two with *ZP2* mutations, and three with *ZP4* mutations had successful pregnancies through Intracytoplasmic sperm injection (ICSI) from ZFO or ZTO. A prediction model of pregnancy outcome in patients with *ZP* mutation was constructed to assess the chance of pregnancy with the area under the curve (AUC) of 0.898. The normalized confusion matrix showed the true positive rate was 1.00 and the true negative rate was 0.38.

**Conclusion:**

Phenotypes in patients with *ZP* mutations might be associated with mutation sites or the degree of protein dysfunction. Successful pregnancy outcomes could be achieved in some patients with identified *ZP* mutations. Clinical pregnancy prediction model based on *ZP* mutations and clinical characteristics will be helpful to precisely evaluate pregnancy chance and provide references and guidance for the clinical treatment of relevant patients.

**Supplementary Information:**

The online version contains supplementary material available at 10.1186/s12958-022-01046-6.

## Introduction

Female infertility has become an increasingly prominent problem affecting approximately 50 million women worldwide [1]. Regular ovulation and healthy oocytes are the significant determinants of female fertility. Defects in this process usually lead to fertilization failure, as well as zygotic or embryonic arrest [2]. The anomalies of the oocyte are challenging to discover via routine examinations. Still, now with the development of in vitro fertilization (IVF) as a treatment for infertility, we have been able to retrieve oocytes and discover their defects in vitro [3].

In mammals, regular fertilization depends on the structural and functional integrity of the zona pellucida (ZP), an extracellular matrix surrounding oocytes [4]. The human ZP contains four glycoproteins (hZP1–4), only three of which (mZP1–3) are found in the mouse due to the pseudogenisation of *ZP4* [5]. ZP glycoproteins contain multiple functional domains and are synthesized, processed, secreted, and assembled into long, cross-linked fibrils by growing oocytes [6, 7]. It’s reported that ZP glycoproteins play a critical role in oogenesis and ensure specific recognition of sperm-egg binding, inducing sperm exocytosis and the acrosome reaction [4, 8–10]. ZP glycoproteins also have a role in preventing polyspermy and protecting the growing embryo till implantation [4, 11].

Based on the crucial role of ZP, morphological assessment of ZP has become a meaningful way to determine the quality of oocytes in assisted reproductive technology (ART) [12]. ZP dysmorphology can include extracellular abnormalities such as a dark ZP, an irregularly shaped ZP, or the absence of a ZP, which contains an incidence of 2–5% of all oocytes [13, 14]. Previous reports suggested that abnormalities in the ZP of oocytes may result in poor pregnancy outcomes in vitro, but the mechanism of ZP dysmorphology is rarely reported in the literature [15]. In this context, some early evidence suggested that there might be a causal relationship between gene sequence variations (GSV) in h*ZP* genes and female fertility [16–18]. Therefore, understanding the genetic mechanism behind abnormal ZP formation and maintenance is significant for the success rate of IVF.

In order to fully understand the effect of *ZP* mutations on female infertility, we collected all the case studies related to GSV in h*ZP1–4* up to June 30, 2022. In addition, we discussed the relationship between mutation types and clinical phenotypes, and established a model to predict pregnancy outcomes of patients with *ZP* mutations. The aim of our study was to assess the genetic mechanism of ZP defects, and to provide more reference and guidance for the clinical ART treatment and clinical outcome prediction of relevant patients.

## Methods

### Search strategy

Cases of patients with *ZP* mutations were identified through a literature review according to the Preferred Reporting Items for Systematic reviews and Meta-Analyses (PRISMA) guideline [19]. The search was completed from January 1, 2014 to June 30, 2022 from the PubMed database using the following keywords: (“Zona Pellucida”[Mesh] OR “Zona pellucida”[All Fields]) AND (“Mutation”[Mesh] OR “Mutation”[All Fields] OR “Variant”[All Fields]). The search was restricted to publications with English titles and abstracts. Case reports and case series were included if they contained: (1) The reported new mutations need to meet the needs of previously unreported public databases, such as low allele frequencies in ExAC and gnomAD databases, mutations in exons or splice sites, and computer predictions of pathogenicity or possible pathogenicity. (2) The information of the probands is complete, including a full genetic pedigree, detailed medical records of the patients receiving ART, as well as fertilization and pregnancy outcomes. The references of publications were reviewed to identify additional cases. Publications and cases were excluded if *ZP* mutations were identified in other diseases instead of infertility or existed clear controversy. For each publication the following data were extracted: patient age and duration of infertility, ART treatment process, *ZP* mutations information, phenotypes and pregnancy outcomes.

### Data analysis

All tests were performed using the statistical software R (http://www.r-project.org/). *P* < 0.05 was considered statistically significant. The idea of Bayes classifier is to calculate the joint probability distribution based on the assumption of feature independence. For a given input feature, the Bayes theorem is used to calculate the maximum posterior probability output category. In the Bayes classifier, instances of known classes are learned, a probabilistic model of attributes is established, and the model is used to predict the classification of new instances. In the small sample data set, the naive Bayes classifier still has a good performance, and the result calculation depends on the prior probability. Considering that the data in this study was less and the prior probability was easy to calculate, the naive Bayes classification method was chosen. In addition, in our previous study, the naive Bayes classifier has been applied to predict clinical pregnancy outcomes for transferred blastocysts with good prediction effect [20]. Therefore, based on mutation and clinical characteristics of patients, the naive Bayes classifier was used to predict clinical pregnancy outcomes for patients with *ZP* mutations, and the code editing was conducted in python (https://www.python.org/).

### Supplementary information collection

To acquire detailed and long-term pregnancy outcomes, we emailed all authors of the included literature. The emails contained whether there were new IVF attempts or successful pregnancies achieved in the identified patients after the publication of the literature.

## Results

### Literature review

**Fig. 1:**
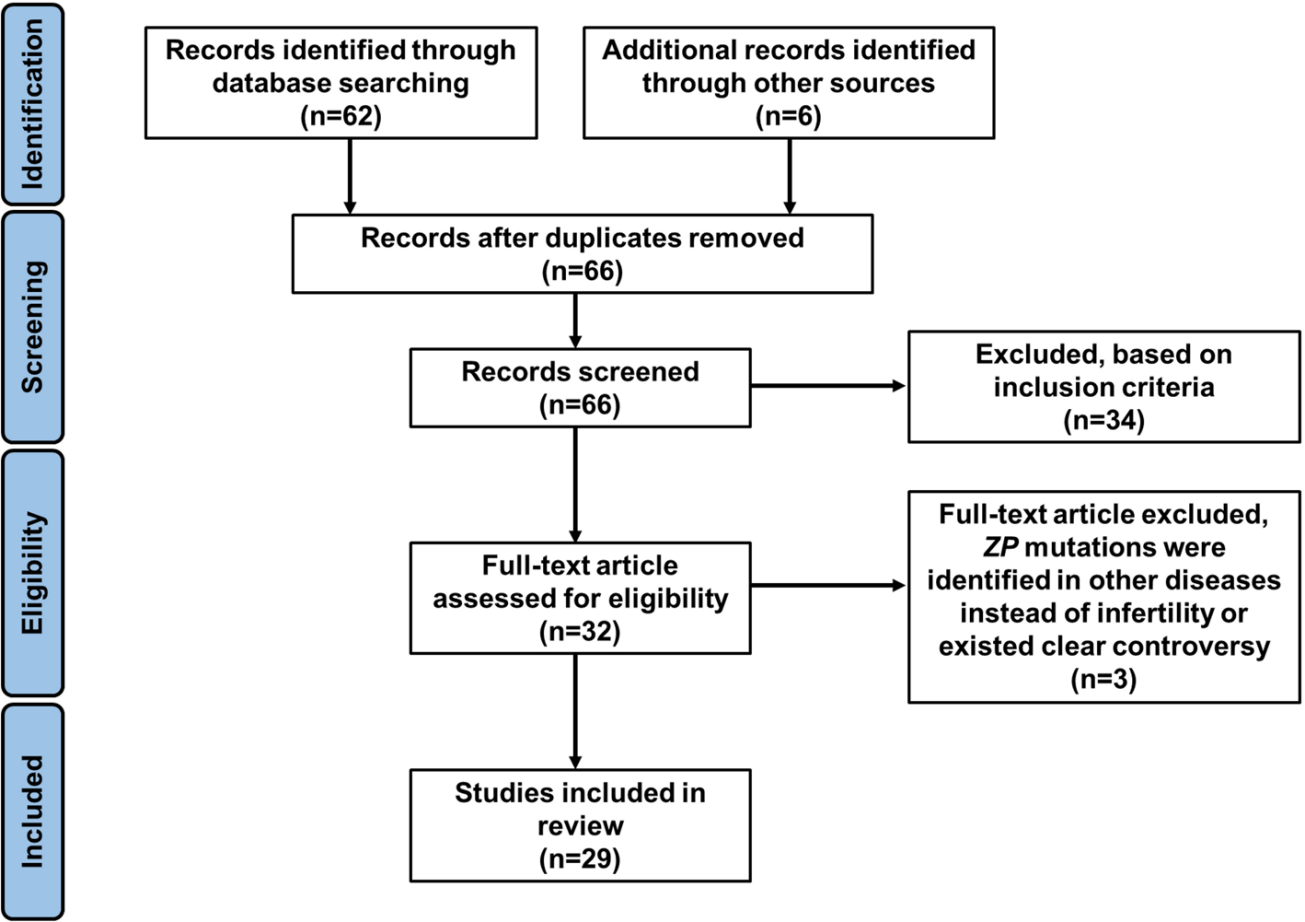
PRISMA flowchart detailing the selection of publications for this literature review of identified *ZP* mutations

A PRISMA flowchart detailing the selection of publications is shown in Fig. 1. A total of 68 studies were identified through the keyword search. After duplicates were removed, 66 studies were screened via title and abstract for inclusion in the full-text review. Thirty-two articles underwent full-text review. Three studies were excluded because *ZP* mutations were identified in other diseases instead of infertility or existed clear controversy [21]. A total of 29 publications were included in the final analysis. Table 1 shows the relevant information of the included literature.

**Table 1:** Summary of information of the included literature

Authors, Year published	Mutate *ZP* gene(s)	Number of cases	Phenotype(s)	Successful pregnancies
Huang et al., 2014 [22]	*ZP1*	2	ZFO	N
Chen et al., 2017 [23]	*ZP3*	6	EFS	N
Yang et al., 2017 [24]	*ZP1, ZP2*	4	degenerated, ZFO, ZTO	N
Dai, Chen, et al., 2019 [25]	*ZP1*	5	EFS	N
Dai, Hu, et al., 2019 [26]	*ZP2*	2	ZTO	Y
Sun et al., 2019 [27]	*ZP1*	2	EFS	N
Yuan et al., 2019 [28]	*ZP1*	1	EFS	N
Zhou et al., 2019 [29]	*ZP1, ZP2, ZP3*	7	ZTO, ZFO, EFS	Y
Cao et al., 2020 [12]	*ZP1, ZP3*	2	ZFO	Y
Chu et al., 2020 [30]	*ZP1*	1	ZFO	Y
Liu et al., 2020 [31]	*ZP1*	1	EFS	N
Zhang et al., 2020 [32]	*ZP1*	1	ZFO	N
Luo et al., 2020 [33]	*ZP1, ZP2*	3	ZTO, ZFO. EFS	N
Metwalley et al., 2020 [34]	*ZP1*	1	ZFO	Y
Okutman et al., 2020 [35]	*ZP1*	3	immature, ZFO	N
Xu et al., 2020 [36]	*ZP1*	1	EFS	N
Chen et al., 2021 [37]	*ZP3*	1	EFS	N
Sun et al., 2021 [38]	*ZP2*	1	EFS	N
Wang et al., 2021 [39]	*ZP1*	1	EFS	N
Wu et al., 2021 [40]	*ZP1*	5	EFS	N
Yang et al., 2021 [41]	*ZP1, ZP2, ZP3*	18	immature, degenerated ZFO, EFS,	N
Zhang et al., 2021 [42]	*ZP3*	1	ZFO	N
Wei et al., 2022 [43]	*ZP4*	3	ZTO	Y
Hou et al., 2022 [44]	*ZP2*	3	ZTO, ZFO	N
Huo et al., 2022 [45]	*ZP1, ZP2, ZP3*	3	immature, ZFO, EFS	N
Jia et al., 2022 [46]	*ZP2, ZP3*	3	ZTO, EFS	N
Shen et al., 2022 [47]	*ZP2*	3	EFS	N
Zhang et al., 2022 [48]	*ZP3*	2	EFS	N
Zou et al., 2022 [49]	*ZP1*	1	EFS	N

*EFS* empty follicle syndrome, *ZFO* ZP-free oocyte, *ZTO* ZP-thin oocyte, *N* NO, *Y* YES

### Identified *ZP* mutations

**Fig. 2:**
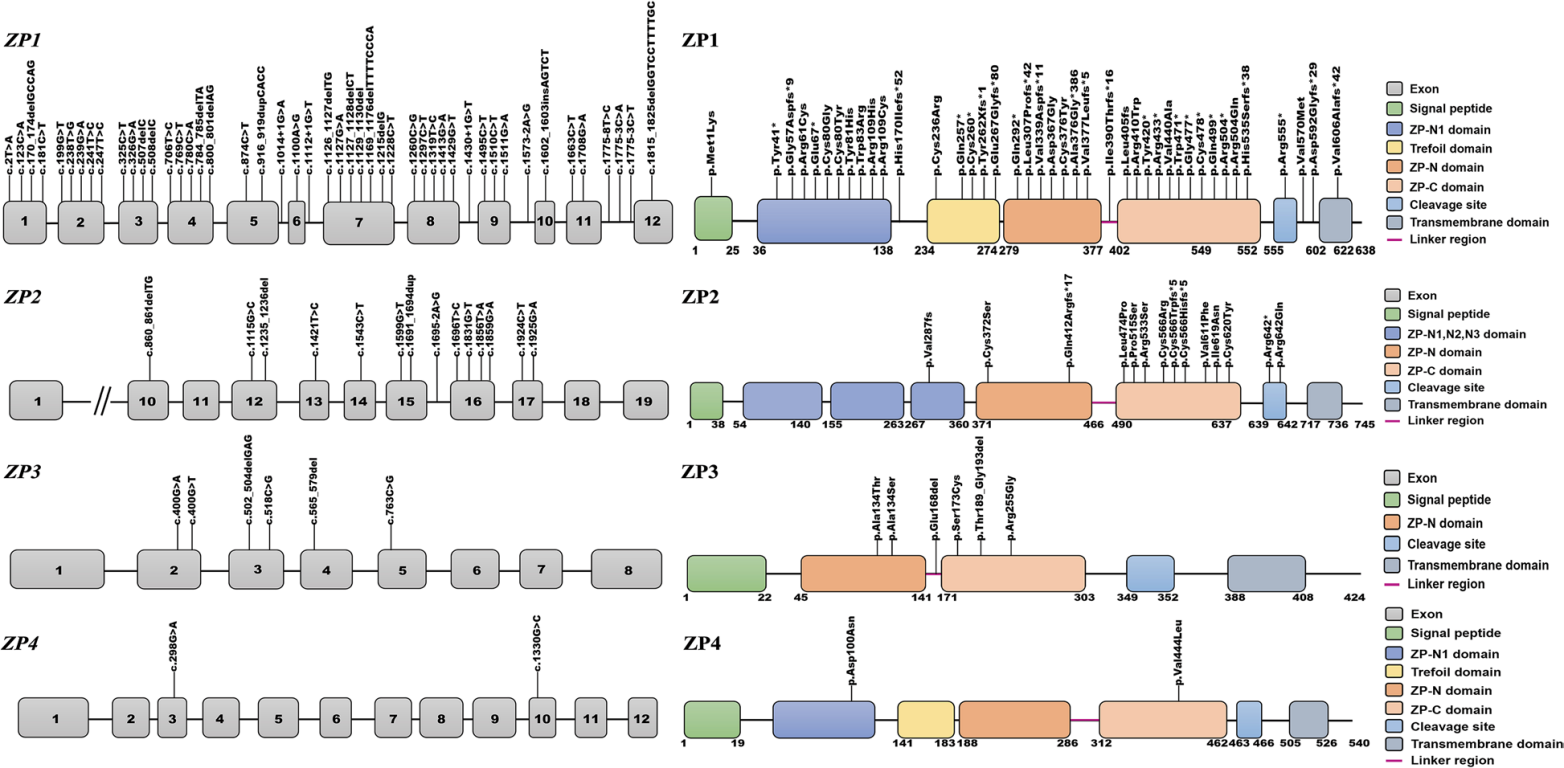
Mutations in *ZP* genes and ZP proteins

A total of 47 *ZP1* mutations have been reported in 19 pieces of literature, most of which are exon mutations, and only seven are intron splicing mutations. Mutations are distributed in all 12 exons of *ZP1* gene. Corresponding protein variants are distributed in all seven functional domains of ZP1 protein, including 13 nonsense mutations, 16 missense mutations, seven splicing mutations and 11 frameshift mutations (Fig. 2A). Their genotypes are homozygous, heterozygous, or compound heterozygous, and are inherited in autosomal dominant (AD) or autosomal recessive (AR) manners, which cause different phenotypes like empty follicle syndrome (EFS), ZP-free oocytes (ZFO), degenerated or immature oocytes (Supplement Table 1).

A total of 14 *ZP2* mutations have been reported in 10 pieces of literature, and only 1 is an intron splicing mutation, all the others are exon mutations. Mutations are distributed in exons 10,12,15 and 17. Corresponding protein variants are distributed in additional N-terminal ZP domain 3 (ZP-N3), ZP domain (ZPD), and consensus furin cleavage-site (CFCS), including one nonsense mutation, nine missense mutations, one splicing mutation and three frameshift mutations (Fig. 2B). Their genotypes are homozygous, heterozygous or compound heterozygous, and are inherited in AD or AR manners, which cause different phenotypes like EFS, ZFO, ZP-thin oocytes (ZTO), degenerated or immature oocytes (Supplement Table 2).

A total of 6 *ZP3* gene mutations have been reported in 9 pieces of literature, all of them are exon mutations, distributed in exon 2 to exon 5, and corresponding protein variants are all distributed in ZPD, including three missense mutations and two frameshift mutations (Fig. 2C). Their genotypes are homozygous, heterozygous, or compound heterozygous, and are all inherited in AD manner, which causes EFS or ZFO (Supplement Table 3).

A total of 2 *ZP4* mutations have been reported in 1 piece of literature, all of them are exon mutations, distributed in exon 3 and 10, and corresponding protein variants are distributed in additional N-terminal ZP domain 1 (ZP-N1) and ZPD, both of them are missense mutations (Fig. 2D). They are both heterozygous and inherited in an AD manner, resulting in ZTO (Supplement Table 4).

### Clinical characteristics, phenotypes, and pregnancy outcomes of patients

Forty-eight primary infertile women with *ZP1* mutations were reported in the literature, aged from 23 to 38 years old, with a history of infertility between 2 and 11 years. Of these patients, 37 showed EFS, degenerated or immature oocytes during different ART cycles, and all canceled the cycles (Table 2). ZFO was collected from 11 patients with different ART cycles; four were successfully fertilized and three were successfully conceived, all by intracytoplasmic sperm injection (ICSI) (Supplement Table 5). One patient carried AD heterozygous mutation c.326G > A; in her only ART cycle, eight cumulus-oocyte complexes (COCs) were collected by gonadotropin-releasing hormone agonist protocol, and eight oocytes were isolated, of which five degenerated and the remaining three were ZP-free mature oocytes. Two mature oocytes were successfully fertilized and developed to blastocyst state, while one oocyte degraded after ICSI. The patient finally successfully delivered an Apgarscore10 baby [12]. Another patient carried compound heterozygous mutation c.1100A > G and c.1215delG. The first two IVF cycles failed and the third cycle was changed to ICSI. After 2 cycles of oocyte extraction, a ZFO was obtained and fertilized successfully by ICSI. A 6 BC embryo was developed and cryopreserved. After another failure in egg retrieval, the frozen-thawed embryo transfer was performed and the patient delivered a healthy baby by planned cesarean section at 38 weeks of pregnancy [30]. The third patient carried homozygous missense mutation c.706 T > C; after the first failed ART, diagnostic intracytoplasmic sperm injection (D-ICSI) was used in the normal menstrual cycle to explore the feasibility of embryo development, then a new ART cycle was started to obtain 8 ZFO, 6 of them were fertilized successfully via ICSI, and finally delivered a healthy baby at 38 weeks of gestation by cesarean section. Additionally, we get the information from the author’s email that she is currently pregnant with twins through ICSI with the modified protocol [30, 34].

**Table 2:** Summary of phenotypes and pregnancy outcomes in patients with ZP mutations

	Patients	Phenotype	Fertilization	Embryos	Pregnancy
*ZP1*	48	EFS/degenerated/immature(37)	N	N	N
		ZFO(11)	Y	Y	Y(3)
*ZP2*	20	EFS/degenerated/immature(9)	N	N	N
		ZFO/ZTO(11)	Y	Y	Y(2)
*ZP3*	16	EFS(11)	N	N	N
		ZFO/ZTO(5)	N	N	N
*ZP4*	3	ZTO(3)	Y	Y	Y(3)

A total of 87 patients with *ZP* mutations were included in the study. They showed different clinical phenotypes, including EFS, ZFO, ZTO, degenerated or immature oocytes. Three patients with *ZP1* mutations, two patients with *ZP2* mutations, and three patients with *ZP4* mutations achieved successful clinical pregnancies. *EFS* Empty Follicular Syndrome, *ZFO* ZP-free Oocyte, *ZTO* ZP-thin Oocyte, *Y* Yes, *N* No

Twenty primary infertile women with *ZP2* mutations were reported in pieces of literature, aged from 25 to 38 years old, with a history of infertility of 3 to 13 years. In different ART cycles, nine patients showed EFS, degenerated or immature oocytes, and the other 11 collected ZTO or ZFO (Table 2). Twelve patients experienced successful fertilization among them, but up to the time of publication of the literature, only two patients had successful pregnancies (Supplement Table 6). Patient with c.1115G > C mutation obtained only one poor quality embryo after four IVF cycles, but this embryo led to a successful pregnancy (specific clinical information was unknown) [29]. In the first cycle of the patient with c.1695-2A > G mutation, all the mature oocytes were divided into the IVF group and the ICSI group. None of the IVF group was fertilized. In the ICSI group, all 8 ZTO were fertilized, and two fresh 8-cell embryos were transferred, but the transfer failed. The remaining embryos formed two blastocysts and were cryopreserved. In the following frozen embryo transfer cycle, two vitrified blastocysts were thawed and transferred (1–4 BC, 1-5 BC), resulting in a singleton pregnancy with an estimated gestational age of 39 weeks [26].

Sixteen primary infertile women with *ZP3* mutations were reported in the literature, aged from 26 to 38 years old, with a history of infertility of 2–11 years. Eleven patients showed EFS; the other five collected ZFO or ZTO in different ART cycles (Table 2). However, until now, no cases of successful pregnancy have been reported (Supplement Table 7).

Three primary infertile women with *ZP4* mutations were reported in the literature, aged from 33 to 34 years old, with a history of infertility of 3 to 5 years (Supplement Table 8). All patients collected ZTO (Table 2). The first patient carried heterozygous mutation c.298G > A; after two failed cycles of IVF, ICSI was performed for the third time, three ZTO were obtained and all of them were fertilized. A six-cell cleavage embryo was adopted to transfer and finally achieved clinical pregnancy. Based on this successful experience, the other two patients carrying c.298G > A or c.1330G > C also achieved clinical pregnancy through ICSI treatment [43].

**Fig. 3:**
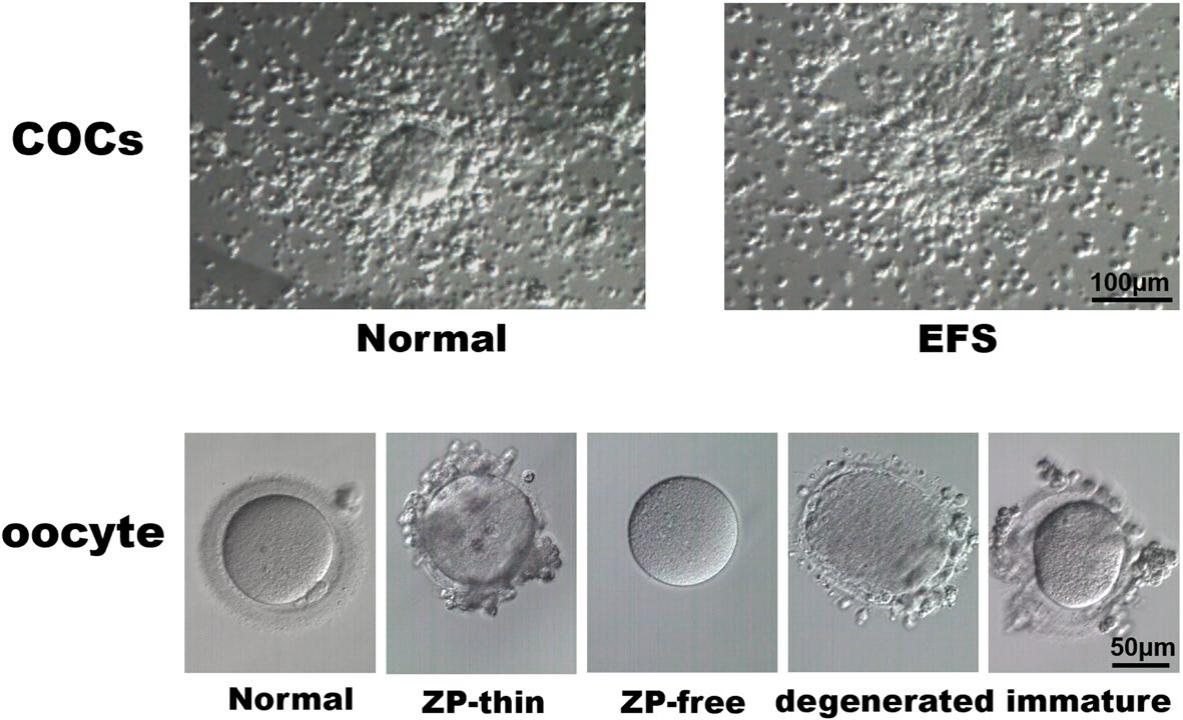
Different phenotypes of patients with *ZP* mutations. Different mutations of *ZP* can cause different clinical phenotypes, including empty follicle syndrome, ZP-thin oocytes, ZP-free oocytes, degenerated oocytes, and immature oocytes. COC, cumulus-oocyte complexes; EFS, empty follicle syndrome

In general, phenotypes of *ZP* mutations reported so far include EFS, ZTO, ZFO degenerated and immature oocytes (Fig. 3). The phenotypes of patients are influenced by the types and location of the mutations. Protein truncation is the most common condition caused by nonsense and frameshift. The earlier the truncated sites appear, the less the protein functional domains are retained, which leads to different degrees of ZP defects. Successful fertilization and pregnancy have been reported in ZTO and ZFO phenotypes, which are less affected by mutations. In addition, successful pregnancy cases have been reported in *ZP1, ZP2,* and *ZP4* mutations, while there has been no report in *ZP3* mutations.

### Correlation analysis between the truncation sites and phenotypic severity

In order to explore whether the difference in phenotypic severity is affected by different truncation sites, we performed a correlation analysis with cases of *ZP1* truncation mutations, which had a relatively large amount of data. Of the 41 *ZP1* truncation mutations (including mutation site truncation and frameshift truncation), 33 cases were phenotyped as EFS/degenerated/immature and eight cases were phenotyped as ZFO. The Levene variance equality test in Rstudio4.1.1 showed *F* = 5.536, *P* = 0.024 < 0.05, therefore independent sample test without assuming equal variance was calculated as *P* = 0.065 > 0.05, indicating no significant difference between the truncation sites of the two phenotypes in the available data.

### Correlation analysis of truncation mutations and EFS

The EFS was found to occur in many cases carrying truncation mutations in the study, so the correlation between mutation types and phenotypes was analyzed in Rstudio4.1.1 for 47 *ZP1* mutation samples, where the mutation types were divided into truncation and substitution, and the phenotypes were divided into EFS and non-EFS. The four-compartment table is shown in Supplement Table 9, where *N* > 40 and T < 5, which needs to be corrected for chi-square values, and the corrected χ^2^ = 2.713, *P* = 0.10 > 0.05, indicating that the difference in the proportion of truncation mutation in EFS and non-EFS samples is not statistically significant in the available data.

### Prediction model of pregnancy outcome

Patients with different mutation types may have different phenotypes and pregnancy outcomes. Therefore, we propose that analyses of information on related mutations and phenotypes of patients may help predict pregnancy outcomes. In this study, a naive Bayes model was constructed based on the analysis of 86 samples to predict clinical pregnancy outcomes by using four characteristics: the mutated *ZP* gene, the protein domain where the mutation is located, the type of mutation, and the clinical phenotype of the patient. The study used one-hot coding, coding non-pregnancy as 1 and pregnancy as 0.

**Fig. 4:**
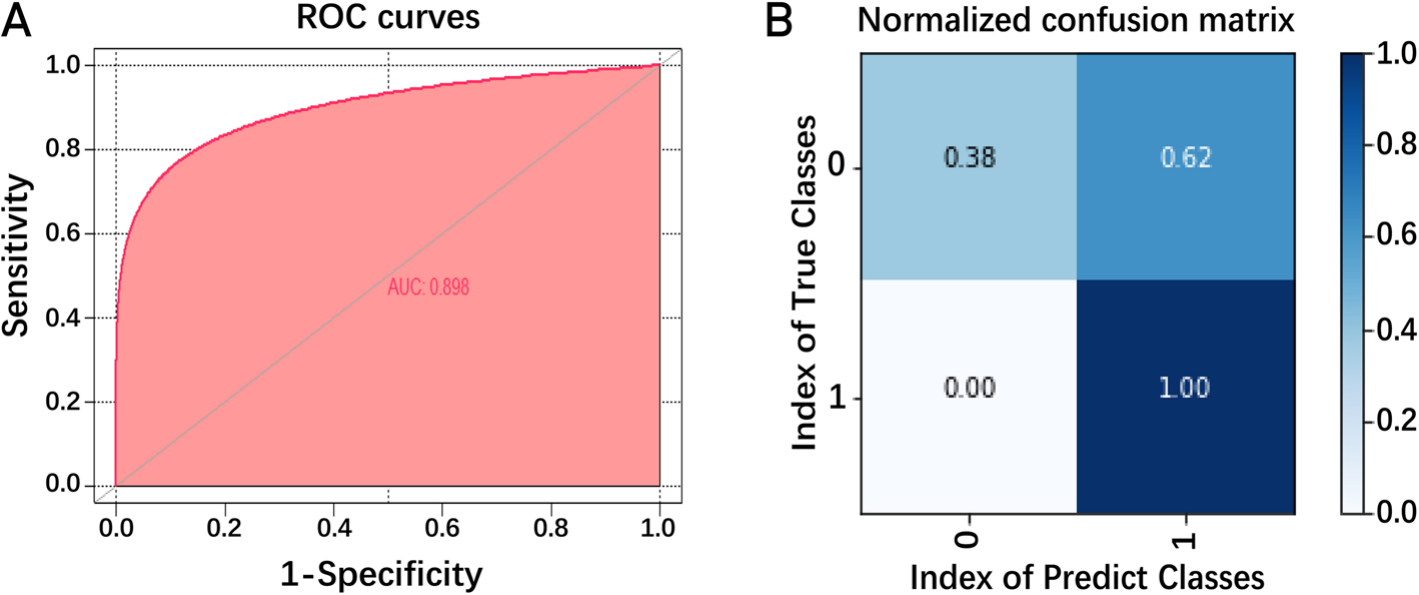
Prediction model of pregnancy outcome. **A.** The receiver operating characteristic (ROC) curves of a mixture of four features, including the mutated *ZP* gene, the protein domain where the mutation is located, the type of mutation, and the clinical phenotype of the patient. The area under the curve (AUC) is 0.898. **B.** The normalized confusion matrix shows the true positive rate is 1.00 and the true negative rate is 0.38. The study used one-hot coding, coding non-pregnancy as 1 and pregnancy as 0

The study used Python3.8.8 to construct the prediction model, and the performance of the constructed model is shown in Supplement Table 10. As shown in the table, the accuracy, precision, recall and F1 of the model are high. The receiver operating characteristic (ROC) curves illustrates the efficiency of mutation-related features in predicting pregnancy outcome. Figure 4A shows an area under the curve (AUC) of 0.898, indicating the model has good classification effectiveness. The normalized confusion matrix shows the true positive rate is 1.00 and the true negative rate is 0.38 (Fig. 4B).

## Discussion

ZP is an extracellular glycoproteinaceous coat surrounding oocytes and plays a vital role during oogenesis, fertilization, and preimplantation development [4, 5]. Failure to assemble a normal ZP around growing oocytes during oogenesis results in female infertility. According to the collected literature, gene sequence variations in h*ZP* due to missense, nonsense, splicing or frameshift have been reported as the crucial cause of ZP formation defects and female fertility.

The *ZP* genes have previously been studied in mice. *ZP1*-null mice showed oocytes with loosely organized ZP; the mice were less fecund but fertile [50]. *ZP2*-null or *ZP3*-null mice produced eggs without a ZP, as well as infertility [51, 52]. But human patients with *ZP* mutations have a variety of phenotypes, ranging from a thin or absent ZP to the most severe manifestations of EFS (Fig. 3). The phenotypic differences between mice and humans may be due to the differences in the number and transport mechanism of human and mouse ZP glycoproteins, and the differences in sensibility to ZP defects [53]. Another crucial cause of phenotypic variability may be attributed to the different positions of the variants and the associated structural changes of the protein.

**Fig. 5:**
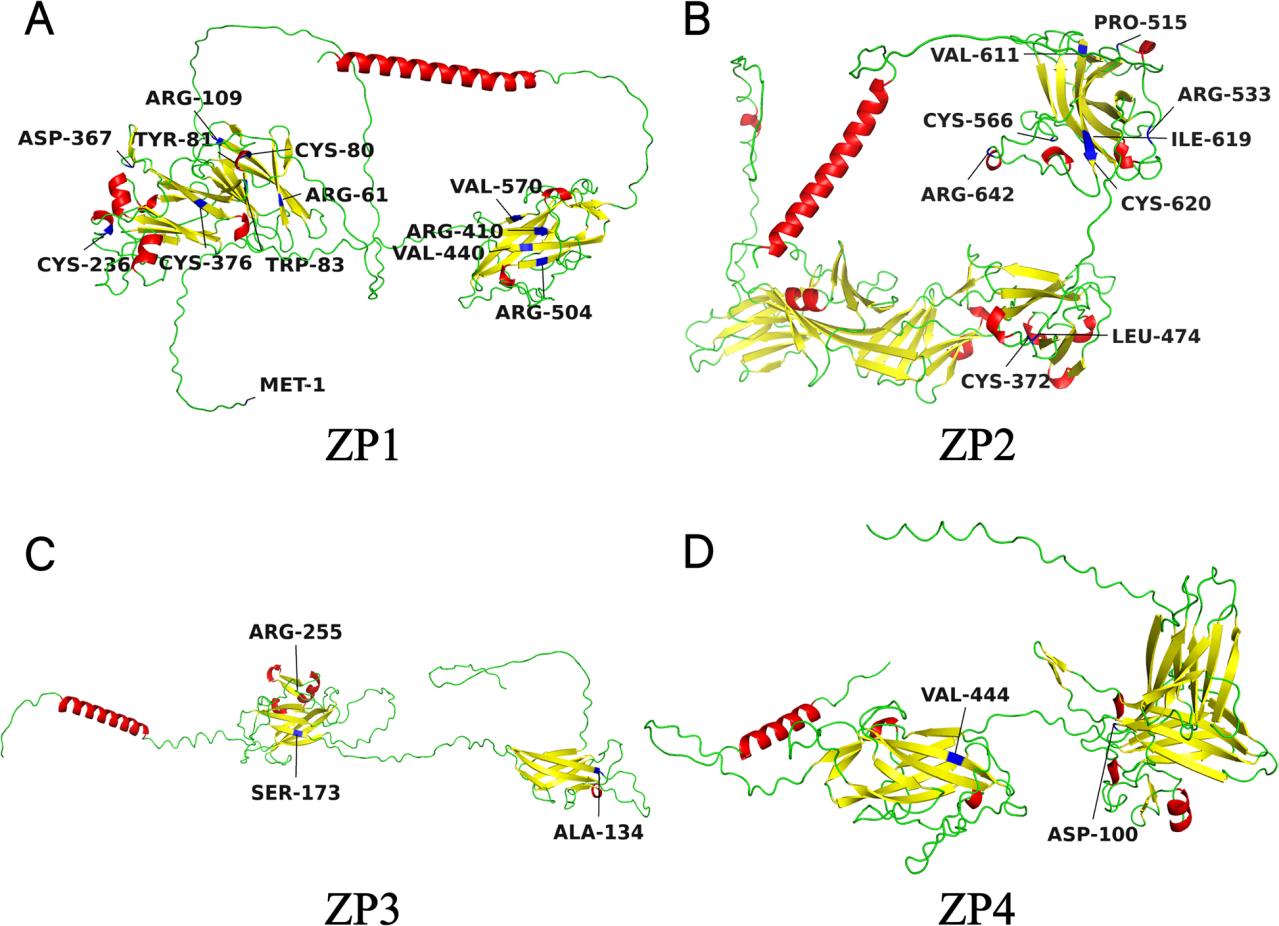
Three dimensional models of ZP proteins with missense mutations. The structures of ZP proteins are predicted by AlphaFold from AlphaFold Protein Structure Database (https://alphafold.ebi.ac.uk/). Alpha helixes are shown in red, beta folds are shown in yellow, loops are shown in green. Amino acid residues with missense mutations are shown in blue

In terms of the types of mutation, missense results in the substitution of corresponding amino acid residues, which may affect the function of related ZP domains (Fig. 5). The N-terminal hydrophobic signal sequence (SS) can target ZP to the secretory pathway through co-translational import into the endoplasmic reticulum and which ultimately gets cleaved from the mature proteins by signal peptidase present in the oocytes [5]. Mutations in this domain may damage the expression of ZP [31]. ZPD plays an essential role in the secretion of ZP and the polymerization of ZP into filaments; mutations mainly occur in this region [3, 54]. Dysfunction of ZPD caused by mutation will not only influence the secretion of ZP but also primarily affect the interaction between ZP proteins [12, 29, 37]. Immediately after ZPD, all four human ZP glycoproteins have CFCS. Proteolytic cleavage at CFCS by proprotein convertase enzyme is critical for the secretion and assembly of ZP proteins [55]. Mutations in this domain show secretion defects and altered protein modification [41]. Downstream of CFCS, a hydrophobic transmembrane domain (TMD) and a short cytoplasmic tail (CT) are present in all four human ZP glycoproteins [3]. TMD can prevent ZP proteins’ premature intracellular interaction and thus plays a vital role in incorporating ZP proteins into the ZP matrix [56]. Dysfunction in this domain may cause intracellular sequestration and aggregation of ZP proteins [26, 40]. Besides ZPD, additional N-terminal ZP1 domain 1 (ZP1-N1) is another domain where mutations mainly occurred. Recent research suggests that ZP1-N1 cross-link formation is essential for the assembly or maintenance of the hZP and fertility, which shows that mutations in this domain may affect the cross-linking function of ZP1 and then influence the formation of the ZP matrix [12, 57].

As for nonsense and frameshift mutations, which usually cause the premature emergence of stop codons, the severity of clinical manifestations may depend on the residual function in the mutant ZP protein. We hypothesize that the earlier the truncation site appears, the less protein function remains, which may cause a more severe phenotype, with EFS/degeneration as the most severe phenotype reported in patients with *ZP* mutations. However, correlation analysis shows no significant difference in truncation sites across phenotypes (*P* = 0.065 > 0.05) and no significant difference in the proportion of truncation mutations in EFS and non-EFS (*P* = 0.10 > 0.05), which may be limited by the sample size.

EFS is a condition in which the ovarian response to stimulation and follicular development seems normal but no oocytes are retrieved for fertilization [58]. EFS can be classified as either false EFS (FEFS) or genuine EFS (GEFS). FEFS is mainly caused by pharmacological or iatrogenic problems [59, 60]. However, the estimated prevalence of GEFS is 0.016% among patients who underwent IVF [61, 62]. While some previous studies have suggested that dysfunctional folliculogenesis and ovarian aging are involved in GEFS, more recent efforts implicated substantial genetic factors contributing to GEFS, including mutations in luteinizing hormone/choriogonadotropin receptor (*LHCGR*) [63, 64], the pericentric inversion of chromosome 2, and mutations in *ZP1*, *ZP2,* and *ZP3* as shown above. The phenotypes between mutations in the *LHCGR* and *ZP* genes present differently. Neither COCs nor oocytes could be identified in patients with *LHCGR* mutations, while COCs could be obtained but only degenerated oocytes or no oocytes could be identified in patients with *ZP* mutations (Fig. 3). Thus, some articles propose standardizing the definition of ZP-related EFS as a subtype of EFS characterized by degeneration [39]. In addition, the recovery of a few mature or immature oocytes from mature follicles has been described in some literature as a borderline form of EFS [65–67], which can also appear in *ZP* mutations (Fig. 3).

According to the statistics, mutations in *ZP1* and *ZP3* resulted in the loss of the ZP, and EFS occurred at a high rate (Table 2). This is consistent with the function of the relevant ZP proteins; that is, ZP1 serves to cross-link ZP filaments made up of ZP2–ZP3 dimers [50, 68], and ZP3 is indispensable for the formation of ZP and is also necessary for ovulation and producing the cleaved embryos [52, 69]. Lack of ZP might interfere with the bidirectional communication between oocytes and cumulus cells, then impedes nutritional supplements from cumulus cells leading to oocyte degeneration or increases in the fragility of oocytes during follicular puncture, ultimately resulting in EFS [8, 53, 70]. In addition, patients with *ZP3* mutations have been no successful pregnancies so far (Table 2), which suggests a crucial role of ZP3 in ZP assembly and supports the opinion that the absence of ZP3 cannot be overcome [71]. Mutations in *ZP2* were mainly manifested as ZTO, and the proportion of successfully fertilized oocytes was relatively high via ICSI (Table 2, Supplement Table 6). This may be because ZP2 is mainly involved in sperm recognition and binding, as well as the prevention of polyspermy [72]. Oocytes obtained from patients with *ZP4* mutations were free of ZP and all showed successful fertilization and pregnancy via ICSI, which suggested that *ZP4* variants perhaps do not dramatically affect embryonic development [43].

It’s worth noting that, the severity of patients’ phenotypes may be influenced by genotypes in addition to the degree of residual protein domains. In some patients, the presence of oocytes lacking ZP but not degenerated is likely to be dependent on the residual function of the mutant ZP protein, or the complementary role of one copy of the intact allele in heterozygous patients [36, 39]. Liu et al. used gene editing mice to further study two mutations they found in h*ZP* (despite controversy), and found that either of the heterozygous mutations showed thinner ZP as compared to the oocytes obtained from wild mice. Moreover, it was reported that the mother of the patient carrying the *ZP3* mutant was fertile, while the patient carrying both the *ZP2* and *ZP3* mutants showed much thinner ZP and complete fertilization failure, which suggests that these mutations have a dosage-dependent effect on the formation of ZP [73]. In addition, a case report showed that a frameshift mutation (c.860_861delTG) in *ZP2* was inherited from the patient’s father, and the unaffected older sister who carried the heterozygous mutation alone had a child; only the patient who carried the compound heterozygous mutation is infertile [33]. It indicates that the genotypes of patients with homozygosis, heterozygosis, or compound heterozygosis might also affect phenotypes, and further research is needed.

According to our statistics, three patients with *ZP1* mutations, two patients with *ZP2* mutations, and three patients with *ZP4* mutations had successful pregnancies through ICSI from ZFO or ZTO (except one patient with *ZP2* mutation whose specific clinical information was not available) (Table 2), which suggests patients with *ZP* mutations still have chances of successful pregnancies. In fact, the developmental potential of the ZFO is reported to be similar to the zona intact oocytes in terms of fertilization, culture, and pregnancy outcomes [74]. Studies also show that although the ZP partially functions as a sperm receptor and a protective sheath for developing embryos during the cleavage stage, the ZP maybe not be an essential component for oocyte maturation and embryogenesis in the IVF settings, despite its importance during oocyte assessment and handling [32]. Therefore, for infertile patients with ZP defects or EFS, the possibility of *ZP* gene mutations should be considered, and whole-exome sequencing screening is recommended as a beneficial detective to identify the causes of infertility. What’s more, for patients with identified *ZP* mutations, the treatment plan of the following IVF attempt should consider the phenotype’s severity and the specific *ZP* mutation mode. If oocytes with ZP defects could be obtained from the affected patients, ICSI could be preferentially chosen instead of the traditional IVF, which may improve the probability of successful pregnancy via ART. Metwalley et al. proposed a new method called D-ICSI, an ICSI cycle performed in the natural cycle, to obtain information about embryo development potential before therapeutic ICSI [34]. For patients with the most severe phenotypes of EFS caused by *ZP* gene mutations, the current optimal treatment choice would be egg donation. Based on the evidence reported that the oocytes existed at least progressing normally through the preantral stages of folliculogenesis in EFS [25], a further approach may involve culturing early-stage oocytes in vitro to maturity before they can degenerate, but this treatment requires further study. In addition, for patients continuing to attempt IVF, the use of the pregnancy prediction model for *ZP* mutations is strongly recommended to assess the chances of pregnancy (Fig. 4), so as to provide better fertility counseling and reproductive guidance for patients.

## Conclusion

In general, we summarized the currently reported cases with mutations in *ZP* genes. We found that different phenotypes in patients with *ZP* mutations might be associated with differences in mutation sites or the degree of protein dysfunction. Despite the significant impact on fertility, some patients with *ZP* mutations still have chances of successful pregnancies. Clinical pregnancy prediction model based on *ZP* mutations and clinical characteristics will be helpful to precisely evaluate pregnancy chance and provide references and guidance for the clinical treatment of relevant patients.

## Supplementary Information

**Additional file 1.**

## Data Availability

All data generated or analysed during this study are included in this published article and its supplementary information files.

## Abbreviations

AD: autosomal dominant
AR: autosomal recessive
ART: assisted reproductive technology
AUC: area under the curve
CFCS: consensus furin cleavage-site
COCs: cumulus-oocyte complexes
CT: cytoplasmic tail
D-ICSI: diagnostic intracytoplasmic sperm injection
EFS: empty follicle syndrome
FEFS: false empty follicle syndrome
GEFS: genuine empty follicle syndrome
GSV: gene sequence variations
hZP: human zona pellucida
ICSI: intracytoplasmic sperm injection
IVF: in vivo fertilization
*LHCGR*: luteinizing hormone/choriogonadotropin receptor
mZP: mouse zona pellucida
PRISM: Preferred Reporting Items for Systematic reviews and Meta-Analyses
ROC: receiver operating characteristic
SS: signal sequence
TMD: transmembrane domain
ZFO: ZP-free oocyte
ZP: zona pellucida
ZPD: ZP domain
ZP-N1: N-terminal ZP domain 1
ZP-N3: N-terminal ZP domain 3
ZP1-N1: N-terminal ZP1 domain 1
ZTO: ZP-thin oocyte
