# Supplementary material for: Can successful pregnancy be achieved and predicted from patients with identified ZP mutations? A literature review
Source: Reprod Biol Endocrinol. 2022 Dec 8;20:166. doi: 10.1186/s12958-022-01046-6 (PMC9730648; doi:10.1186/s12958-022-01046-6)
Supplement: Supplementary file 1 — Additional file 1. [file 12958_2022_1046_MOESM1_ESM.docx]

| **cDNA change** | **amino acid change** | **Mutation type** | **Genotype** | **Inheritance** | **Phenotype** | **Reference** |
| --- | --- | --- | --- | --- | --- | --- |
| c.1169_1176delTTTTCCCA | p.Ile390Thrfs*16 | frameshift | homozygous | AR | ZFO | [1] |
| c.1413G>A | p.Trp471* | nonsense | heterozygous | AR | degenerated | [2] |
| c.247T>C,  c.1413G>A | p.Trp83Arg,  p.Trp471* | missense,  nonsense | compound heterozygous | AR | degenerated | [2] |
| c.181C>T,  c.1169_1176delTTTTCCCA | p.Arg61Cys,  p.Ile390Thrfs*16 | missense,  frameshift | compound heterozygous | AR | EFS | [3] |
| c.1708G>A | p.Val570Met | missense | homozygous | AR | ZFO | [4] |
| c.1228C>T | p.Arg410Trp | missense | homozygous | AR | EFS | [4] |
| c.507delC | p.His170Ilefs*52 | frameshift | homozygous | AR | EFS | [4] |
| c.1430+1G>T,  c.1775-8T>C | p.Cys478*,  p.Asp592Glyfs*29 | splicing,  splicing | compound heterozygous | AR | ZFO | [4] |
| c.170_174delGCCAG;  c.1169_1176delTTTTCCCA | p.Gly57Aspfs*9,  p.Ile390Thrfs*16 | frameshift,  frameshift | compound heterozygous | AR | EFS | [5] |
| c.1510C>T | p.Arg504* | nonsense | homozygous | AR | EFS | [6] |
| c.1014+1G>A | NA | splicing | homozygous | AR | EFS | [6] |
| c.123C>A,  c.1663C>T | p.Tyr41*,  p.Arg555* | nonsense,  nonsense | compound heterozygous | AR | EFS | [6] |
| c.1129_1130del | p.Val377Leufs*5 | frameshift | homozygous | AR | EFS | [6] |
| c.1573-2A>G,  c.508delC | NA  p.His170Ilefs*52 | splicing,  frameshift | compound heterozygous | AR | EFS | [6] |
| c.1100A>G,  c.1215delG | p.Asp367Gly,  p.Leu405fs | missense,  frameshift | compound heterozygous | AR | ZFO | [7] |
| c.2T>A,  c.1112+1G>T | p.Met1Lys,  p.Val339Aspfs*11 | missense,  splicing | compound heterozygous | AR | EFS | [8] |
| c.326G>A | p.Arg109His | missense | heterozygous | AD | ZFO | [9] |
| c.769C>T | p.Gln257* | nonsense | homozygous | AR | EFS | [10] |
| c.1775-3C>A | NA | splicing | homozygous | AR | ZFO, immature | [11] |
| c.874C>T,  c.1127_1128delCT | p.Gln292*,  p.Ala376Glyfs*386 | nonsense,  frameshift | compound heterozygous | AR | ZFO | [12] |
| c.507delC,  c.239G>A,  c.241T>C | p.His170Ilefs*52 ,  p.Cys80Tyr,  p.Tyr81His | frameshift,  missense,  missense | compound heterozygous | AR | EFS | [13] |
| c.706T>C | p.Cys236Arg | missense | homozygous | AR | ZFO | [14] |
| c.199G>T | p.Glu67* | nonsense | homozygous | AR | EFS | [15] |
| c.1430+1G>T | p.Cys478* | splicing | homozygous | AR | EFS | [16] |
| c.769C>T,  c.1127G>A | p.Gln257*,  p.Cys376Tyr | nonsense,  missense | compound heterozygous | AR | EFS | [16] |
| c.800_801delAG,  c.1169_1176delTTTTCCCA | p.Glu267Glyfs*80,  p.Ile390Thrfs*16 | frameshift,  frameshift | compound heterozygous | AR | EFS | [16] |
| c.1127G>A,  c.325C>T | p.Cys376Tyr,  p.Arg109Cys | missense,  missense | compound heterozygous | AR | EFS | [16] |
| c.199G>T,  c.1815_1825delGGTCCTTTTGC | p.Glu67*,  p.Val606Alafs*42 | nonsense,  frameshift | compound heterozygous | AR | EFS | [16] |
| c.247T>C,  c.784_785delTA | p.Trp83Arg,  p.Tyr262Xfs*1 | missense,  frameshift | compound heterozygous | AR | ZFO | [17] |
| c.238T>G,  c.1260C>G | p.Cys80Gly,  p.Tyr420* | missense,  nonsense | compound heterozygous | AR | EFS | [17] |
| c.1126_1127delTG,  c.1319T>C | p.Val377Leufs*5,  p.Val440Ala | frameshift,  missense | compound heterozygous | AR | ZFO | [17] |
| c.508delC,  c.916_919dupCACC | p.His170Ilefs*52,  p.Leu307Profs*42 | frameshift,  frameshift | compound heterozygous | AR | EFS | [17] |
| c.874C>T | p.Gln292* | nonsense | homozygous | AR | EFS | [17] |
| c.1495C>T | p.Gln499* | nonsense | homozygous | AR | EFS | [17] |
| c.780C>A,  c.1602_1603insAGTCT | p.Cys260*,  p.His535Serfs*38 | nonsense,  frameshift | compound heterozygous | AR | EFS | [17] |
| c.199G>T,  c.1413G>A | p.Glu67*,  p.Trp471* | nonsense,  nonsense | compound heterozygous | AR | EFS | [17] |
| c.247T>C,  c.1413G>A | p.Trp83Arg,  p.Trp471* | missense,  nonsense | compound heterozygous | AR | degenerated, immature | [17] |
| c.1169_1176delTTTTCCCA,  c.1775-3C>T | p.Ile390Thrfs*16,  NA | frameshift,  splicing | compound heterozygous | AR | degenerated | [17] |
| c.1169_1176delTTTTCCCA,  c.1297C>T | p.Ile390Thrfs*16,  p.Arg433* | frameshift,  nonsense | compound heterozygous | AR | EFS | [17] |
| c.2T>A,  c.1429G>T | p.Met1Lys,  p.Gly477* | missense,  nonsense | compound heterozygous | AR | EFS | [17] |
| c.1511G>A | p.Arg504Gln | missense | homozygous | AR | EFS | [17] |
| c.874C>T | p.Gln292* | nonsense | homozygous | AR | EFS | [18] |
| c.1260C>G | p. Tyr420* | nonsense | homozygous | AR | EFS | [19] |

**Supplement Table 1 Summary of *ZP1* mutations**

AR, autosomal recessive; AD, autosomal dominant; EFS, empty follicle syndrome; ZFO, ZP-free oocyte

**Supplement Table 2 Summary of *ZP2* mutations**

| **cDNA change** | **amino acid change** | **Mutation type** | **Genotype** | **Inheritance** | **Phenotype** | **Reference** |
| --- | --- | --- | --- | --- | --- | --- |
| c.1599G>T | p.Arg533Ser | missense | homozygous | AR | degenerated | [2] |
| c.1696T>C | p.Cys566Arg | missense | homozygous | AR | ZFO, ZTO | [2] |
| c.1695-2A>G | p.Cys566Hisfs*5 | splicing | homozygous | AR | ZTO | [20] |
| c.1691_1694dup | p.Cys566Trpfs*5 | frameshift | homozygous | AR | ZTO | [20] |
| c.1115G>C | p.Cys372Ser | missense | homozygous | AR | ZFO,ZTO | [4] |
| c.860_861delTG,  c.1924C>T | p.Val287fs,  p.Arg642* | frameshift,  nonsense | compound heterozygous | AR | ZFO, ZTO | [13] |
| c.1235_1236del | p.Gln412Argfs*17 | frameshift | homozygous | AR | ZTO | [21] |
| c.1925G>A | p.Arg642Gln | missense | heterozygous | AD | EFS | [17] |
| c.1856T>A | p.Ile619Asn | missense | heterozygous | AD | immature, degenerated | [17] |
| c.1543C>T | p.Pro515Ser | missense | heterozygous | AR/AD | ZFO, immature | [18] |
| c.1925G>A | p.Arg642Gln | missense | heterozygous | AD | EFS | [22] |
| c.1695-2A>G,  c.1831G>T | NA,  p.Val611Phe | splicing,  missense | compound heterozygous | AR | ZTO | [23] |
| c.1695-2A>G,  c.1924C>T | NA,  p.Arg642* | splicing,  nonsense | compound heterozygous | AR | EFS | [23] |
| c.1859G>A | p.Cys620Tyr | missense | heterozygous | AR/AD | ZTO, ZFO | [24] |
| c.1421T>C | p.Leu474Pro | missense | heterozygous | AD | ZTO | [24] |

AR, autosomal recessive; AD, autosomal dominant; EFS, empty follicle syndrome; ZFO, ZP-free oocyte; ZTO, ZP-thin oocyte

| **cDNA change** | **amino acid change** | **Mutation type** | **Genotype** | **Inheritance** | **Phenotype** | **Reference** |
| --- | --- | --- | --- | --- | --- | --- |
| c.400G>A | p.Ala134Thr | missense | heterozygous | AD | EFS | [25] |
| c.763C>G | p.Arg255Gly | missense | heterozygous | AD | ZFO | [4] |
| c.400G>A | p.Ala134Thr | missense | heterozygous | AD | ZFO | [9] |
| c.518C>G | p.Ser173Cys | missense | heterozygous | AD | ZFO | [26] |
| c.400G>A | p.Ala134Thr | missense | heterozygous | AD | EFS | [17] |
| c.565_579del | p.Thr189_Gly193del | frameshift | heterozygous | AD | EFS | [27] |
| c.502_504delGAG | p.Glu168del | frameshift | heterozygous | AD | EFS | [28] |
| c.400G>A | p.Ala134Thr | missense | heterozygous | AD | ZFO, immature | [18] |
| c.400G>T | p.Ala134Ser | missense | heterozygous | AD | ZTO, immature | [23] |

**Supplement Table 3 Summary of *ZP3* mutations**

AD, autosomal dominant; EFS, empty follicle syndrome; ZFO, ZP-free oocyte

**Supplement Table 4 Summary of *ZP4* mutations**

| **cDNA change** | **amino acid change** | **Mutation type** | **Genotype** | **Inheritance** | **Phenotype** | **Reference** |
| --- | --- | --- | --- | --- | --- | --- |
| c.298G>A | p.Asp100Asn | missense | heterozygous | AD | ZTO | [29] |
| c.1330G>C | p.Val444Leu | missense | heterozygous | AD | ZTO | [29] |

AD, autosomal dominant; ZTO, ZP-thin oocyte

| **Patient** | **Duration of infertility(y)** | **cDNA change** | **ART cycles** | **Phenotype** | **2PN** | **Pregnancy outcome** | **Reference** |
| --- | --- | --- | --- | --- | --- | --- | --- |
| *ZP1*-1 | 6 | c.1169_1176delTTTTCCCA | 4 | ZFO | 0 | G | [1] |
| *ZP1*-2 | 2 | c.1169_1176delTTTTCCCA | 1 | ZFO | 0 | G | [1] |
| *ZP1*-3 | 10 | c.247T>C,c.1413G>A | 2 | degenerated | 0 | G | [2] |
| *ZP1*-4 | 5 | c.1413G>A | 3 | degenerated | 0 | G | [2] |
| *ZP1*-5 | 4 | c.181C>T, c.1169_1176delTTTTCCCA | 2 | EFS | 0 | G | [3] |
| *ZP1*-6 | 4 | c.1708G>A | 2 | ZFO | 0 | G | [4] |
| *ZP1*-7 | 6 | c.1228C>T | 3 | EFS | 0 | G | [4] |
| *ZP1*-8 | NA | c.507delC | 1 | EFS | 0 | G | [4] |
| *ZP1*-9 | 6 | c.1430+1G>T, c.1775-8T>C | 3 | ZFO | 0 | G | [4] |
| *ZP1*-10 | 3 | c.170_174delGCCAG, c.1169_1176delTTTTCCCA | 2 | EFS | 0 | G | [5] |
| *ZP1*-11 | 5 | c.170_174delGCCAG, c.1169_1176delTTTTCCCA | 1 | EFS | 0 | G | [5] |
| *ZP1*-12 | 7 | c.1510C>T | 2 | EFS | 0 | G | [6] |
| *ZP1*-13 | 7 | c.1014+1G>A | 1 | EFS | 0 | G | [6] |
| *ZP1*-14 | 11 | c.123C>A, c.1663C>T | 2 | EFS | 0 | G | [6] |
| *ZP1*-15 | 3 | c.1129_1130del | 2 | EFS | 0 | G | [6] |
| *ZP1*-16 | 4 | c.1573-2A>G, c.508delC | 2 | EFS | 0 | G | [6] |
| *ZP1*-17 | 5 | c.1100A>G, c.1215delG | 3 | ZFO | 1 | 1 | [7] |
| *ZP1*-18 | 3 | c.2T>A, c.1112+1G>T | 2 | EFS | 0 | G | [8] |
| *ZP1*-19 | 4 | c.326G>A | 1 | ZFO | 2 | 1 | [9] |
| *ZP1*-20 | 2 | c.769C>T | 1 | EFS | 0 | G | [10] |
| *ZP1*-21 | NA | c.1775-3C>A | 7 | ZFO | 2 | F | [11] |
| *ZP1*-22 | NA | c.1775-3C>A | 1 | immature | 0 | G | [11] |
| *ZP1*-23 | NA | c.1775-3C>A | 1 | immature | 0 | G | [11] |
| *ZP1*-24 | 3 | c.874C>T, c.1127_1128delCT | 2 | ZFO | 0 | G | [12] |
| *ZP1*-25 | 10 | c.507delC, c.239G>A, c.241T>C | 1 | EFS | 0 | G | [13] |
| *ZP1*-26 | 3 | c.507delC, c.239G>A, c.241T>C | 2 | EFS | 0 | G | [13] |
| *ZP1*-27 | 10 | c.706T > C | 2 | ZFO | 6 | 1 | [14] |
| *ZP1*-28 | 7 | c.199G>T | 3 | EFS | 0 | G | [15] |
| *ZP1*-29 | 6 | c.1430+1G>T | 2 | EFS | 0 | G | [16] |
| *ZP1*-30 | 7 | c.769C>T, c.1127G>A | 5 | EFS | 0 | G | [16] |
| *ZP1*-31 | 7 | c.800_801delAG, c.1169_1176delTTTTCCCA | 3 | EFS | 0 | G | [16] |
| *ZP1*-32 | 9 | c.1127G > A, c.325C > T | 2 | EFS | 0 | G | [16] |
| *ZP1*-33 | 10 | c.199G>T, c.1815_1825delGGTCCTTTTGC | 2 | EFS | 0 | G | [16] |
| *ZP1*-34 | 10 | c.247T>C, c.784_785delTA | 2 | ZFO | 0 | G | [17] |
| *ZP1*-35 | 4 | c.238T>G, c.1260C>G | 1 | EFS | 0 | G | [17] |
| *ZP1*-36 | 9 | c.1126_1127delTG, c.1319T>C | 3 | ZFO | 0 | G | [17] |
| *ZP1*-37 | 5 | c.508delC, c.916_919dupCACC | 2 | EFS | 0 | G | [17] |
| *ZP1*-38 | 5 | c.874C>T | 3 | EFS | 0 | G | [17] |
| *ZP1*-39 | 4 | c.1495C>T | 1 | EFS | 0 | G | [17] |
| *ZP1*-40 | 5 | c.780C>A, c.1602_1603insAGTCT | 2 | EFS | 0 | G | [17] |
| *ZP1*-41 | 5 | c.199G>T, c.1413G>A | 3 | EFS | 0 | G | [17] |
| *ZP1*-42 | 10 | c.247T>C, c.1413G>A | 2 | degenerated, immature | 0 | G | [17] |
| *ZP1*-43 | 5 | c.1169_1176delTTTTCCCA,  c.1775-3C>T | 1 | degenerated | 0 | G | [17] |
| *ZP1*-44 | 11 | c.1169_1176delTTTTCCCA, c.1297C>T | 2 | EFS | 0 | G | [17] |
| *ZP1*-45 | 4.5 | c.2T>A, c.1429G>T | 1 | EFS | 0 | G | [17] |
| *ZP1*-46 | 5 | c.1511G>A | 2 | EFS | 0 | G | [17] |
| *ZP1-*47 | 6 | c.874C>T | 2 | EFS | 1 | F | [18] |
| *ZP1-*48 | 2 | c.1260C>G | 1 | EFS | 0 | G | [19] |

**Supplement Table 5 Summary of patients with *ZP1* mutations**

EFS, empty follicle syndrome; ZFO, ZP-free oocyte; G, Not transplant/cancel the operation; F, Transplant without pregnancy; ART, assisted reproductive technology; 2PN: two pronuclear oocyte

**Supplement Table 6 Summary of patients with *ZP2* mutations**

EFS, empty follicle syndrome; ZFO, ZP-free oocyte; ZTO, ZP-thin oocyte; G, Not transplant/cancel the operation; F, Transplant without pregnancy; ART, assisted reproductive technology; 2PN, two pronuclear oocyte

| **Patient** | **Duration of infertility(y)** | **cDNA change** | **ART cycles** | **Phenotype** | **2PN** | **Pregnancy outcome** | **Reference** |
| --- | --- | --- | --- | --- | --- | --- | --- |
| *ZP2*-1 | 5 | c.1599G>T | 3 | degenerated | 0 | G | [2] |
| *ZP2*-2 | 9 | c.1696T>C | 3 | ZFO, ZTO | 0 | G | [2] |
| *ZP2*-3 | 3 | c.1695-2A>G | 1 | ZTO | 8 | 1 | [20] |
| *ZP2*-4 | 7 | c.1691_1694dup | 2 | ZTO | 12 | F | [20] |
| *ZP2*-5 | 10 | c.1115G>C | 3 | ZFO, ZTO | 2 | F | [4] |
| *ZP2*-6 | 6 | c.1115G>C | 4 | NA | 1 | 1 | [4] |
| *ZP2*-7 | 6 | c.860_861delTG, c.1924 C>T | 4 | ZFO, ZTO | 6 | F | [13] |
| *ZP2*-8 | 6 | c.1235_1236del | 2 | ZTO | 3 | G | [21] |
| *ZP2*-9 | 5 | c.G1925A | 3 | EFS | 0 | G | [17] |
| *ZP2*-10 | 7 | c.G1925A | 1 | EFS | 0 | G | [17] |
| *ZP2*-11 | 8 | c.T1856A | 3 | immature, degenerated | 0 | G | [17] |
| *ZP2*-12 | 4 | c.1543C>T | 2 | immature | 3 | G | [18] |
| *ZP2*-13 | 6 | c.1925G>A | 2 | EFS | 0 | G | [22] |
| *ZP2*-14 | 5 | c.1925G>A | 2 | EFS | 0 | G | [22] |
| *ZP2*-15 | 13 | c.1925G>A | 2 | EFS | 0 | G | [22] |
| *ZP2*-16 | 9 | c.1695-2A>G,  c.1831G>T | 1 | ZTO | 10 | F | [23] |
| *ZP2*-17 | 3 | c.1695-2A>G,  c.1924C>T | 2 | EFS | 2 | F | [23] |
| *ZP2*-18 | 3 | c.1859G>A | 1 | ZFO, ZTO | 3 | F | [24] |
| *ZP2*-19 | 5 | c.1421T>C | 5 | ZTO | 9 | F | [24] |
| *ZP2*-20 | 3 | c.1421T>C | 1 | ZTO | 4 | G | [24] |

**Supplement Table 7 Summary of patients with *ZP3* mutations**

| **Patient** | **Duration of infertility(y)** | **cDNA change** | **ART cycles** | **Phenotype** | **2PN** | **Pregnancy outcome** | **Reference** |
| --- | --- | --- | --- | --- | --- | --- | --- |
| *ZP3*-1 | 8 | c.400G>A | 3 | EFS | 0 | G | [25] |
| *ZP3*-2 | 7 | c.400G>A | 3 | EFS | 0 | G | [25] |
| ZP3-3 | 7 | c.400G>A | 3 | EFS | 0 | G | [25] |
| ZP3-4 | 4 | c.400G>A | 2 | EFS | 0 | G | [25] |
| *ZP3*-5 | 9 | c.400G>A | 3 | EFS | 0 | G | [25] |
| *ZP3*-6 | 11 | c.400G>A | 3 | EFS | 0 | G | [25] |
| *ZP3*-7 | 6 | c.763C > G | 3 | ZFO | 0 | G | [4] |
| *ZP3*-8 | 5 | c.400G>A | 3 | ZFO | 0 | G | [9] |
| *ZP3*-9 | 2 | c.518C>G | 2 | ZFO | 0 | G | [26] |
| *ZP3*-10 | 4 | c.400G>A | 2 | EFS | 0 | G | [17] |
| *ZP3*-11 | 7 | c.400G>A | 2 | EFS | 0 | G | [17] |
| *ZP3*-12 | 7 | c.565_579del | 5 | EFS | 0 | G | [27] |
| *ZP3*-13 | 4 | c.502_504delGAG | 3 | EFS | 0 | G | [28] |
| *ZP3*-14 | 1 | c.502_504delGAG | 2 | EFS | 0 | G | [28] |
| *ZP3-*15 | 3 | c.400G>A | 2 | ZFO | 0 | G | [18] |
| *ZP3-*16 | 6 | c.400G>T | 2 | ZTO | 0 | G | [23] |

EFS, empty follicle syndrome; ZFO, ZP-free oocyte; G, Not transplant/cancel the operation; ART, assisted reproductive technology; 2PN, two pronuclear oocyte

**Supplement Table 8 Summary of patients with *ZP4* mutations**

| **Patient** | **Duration of infertility(y)** | **cDNA change** | **ART cycles** | **Phenotype** | **2PN** | **Pregnancy outcome** | **Reference** |
| --- | --- | --- | --- | --- | --- | --- | --- |
| *ZP4*-1 | 5 | c.298G>A | 3 | ZTO | 3 | 1 | [29] |
| *ZP4*-2 | 3 | c.298G>A | 1 | ZTO | NA | 1 | [29] |
| *ZP4*-3 | 4 | c.1330G>C | 1 | ZTO | NA | 1 | [29] |

ZTO, ZP-thin oocyte; ART, assisted reproductive technology; 2PN, two pronuclear oocyte

**Supplement Table 9 The four-compartment table of different phenotypes and different mutation types**

|  | **EFS** | **non-EFS** | **total** |
| --- | --- | --- | --- |
| **truncation** | 27 | 14 | 41 |
| **substitution** | 3 | 3 | 6 |
| **total** | 27 | 17 | 47 |

EFS, empty follicle syndrome

**Supplement Table 10 Evaluation of prediction model**

| **Classifier** | **Accuracy** | **Precision** | **Recall** | **F1** |
| --- | --- | --- | --- | --- |
| NB | 0.942 | 0.945 | 0.942 | 0.930 |

NB, Naive Bayes

**Supplement list:**

1. Huang H-L, Lv C, Zhao Y-C, Li W, He X-M, Li P, et al. Mutant ZP1 in Familial Infertility. N Engl J Med. 2014;370(13):1220-6.

2. Yang P, Luan X, Peng Y, Chen T, Su S, Zhang C, et al. Novel zona pellucida gene variants identified in patients with oocyte anomalies. Fertil Steril. 2017;107(6):1364-9.

3. Yuan P, Li R, Li D, Zheng L, Ou S, Zhao H, et al. Novel mutation in the ZP1 gene and clinical implications. J Assist Reprod Genet. 2019;36(4):741-7.

4. Zhou Z, Ni C, Wu L, Chen B, Xu Y, Zhang Z, et al. Novel mutations in ZP1, ZP2, and ZP3 cause female infertility due to abnormal zona pellucida formation. Hum Genet. 2019;138(4):327-37.

5. Sun L, Fang X, Chen Z, Zhang H, Zhang Z, Zhou P, et al. Compound heterozygous ZP1 mutations cause empty follicle syndrome in infertile sisters. Hum Mutat. 2019;40(11):2001-6.

6. Dai C, Chen Y, Hu L, Du J, Gong F, Dai J, et al. ZP1 mutations are associated with empty follicle syndrome: evidence for the existence of an intact oocyte and a zona pellucida in follicles up to the early antral stage. A case report. Hum Reprod. 2019;34(11):2201-7.

7. Chu K, He Y, Wang L, Ji Y, Hao M, Pang W, et al. Novel ZP1 pathogenic variants identified in an infertile patient and a successful live birth following ICSI treatment. Clin Genet. 2020;97(5):787-8.

8. Liu M, Shen Y, Zhang X, Wang X, Li D, Wang Y. Novel biallelic loss-of-function variants in ZP1 identified in an infertile female with empty follicle syndrome. J Assist Reprod Genet. 2020;37(9):2151-7.

9. Cao Q, Zhao C, Zhang X, Zhang H, Lu Q, Wang C, et al. Heterozygous mutations in ZP1 and ZP3 cause formation disorder of ZP and female infertility in human. J Cell Mol Med. 2020;24(15):8557-66.

10. Xu Q, Zhu X, Maqsood M, Li W, Tong X, Kong S, et al. A novel homozygous nonsense ZP1 variant causes human female infertility associated with empty follicle syndrome (EFS). Molecular Genetics & Genomic Medicine. 2020;8(7).

11. Okutman O, Demirel C, Tulek F, Pfister V, Buyuk U, Muller J, et al. Homozygous Splice Site Mutation in ZP1 Causes Familial Oocyte Maturation Defect. Genes. 2020;11(4).

12. Zhang Z, Shangguan T, Li Y, He W. Loss of zona pellucida in oocytes due to compound heterozygous variants of ZP1 gene. Zhonghua yi xue yi chuan xue za zhi = Zhonghua yixue yichuanxue zazhi = Chinese journal of medical genetics. 2020;37(7):789-91.

13. Luo G, Zhu L, Liu Z, Yang X, Xi Q, Li Z, et al. Novel mutations in ZP1 and ZP2 cause primary infertility due to empty follicle syndrome and abnormal zona pellucida. J Assist Reprod Genet. 2020;37(11):2853-60.

14. Metwalley A, Brasha N, Esteves SC, Fawzy M, Brasha H, Hellani A, et al. Role of diagnostic intracytoplasmic sperm injection (ICSI) in the management of genetically determined zona pellucida-free oocytes during in vitro fertilization: a case report. Zygote. 2020;28(6):519-23.

15. Wang J, Yang X, Sun X, Ma L, Yin Y, He G, et al. A novel homozygous nonsense mutation in zona pellucida 1 (ZP1) causes human female empty follicle syndrome. J Assist Reprod Genet. 2021;38(6):1459-68.

16. Wu L, Li M, Yin M, Ou Y, Yan Z, Kuang Y, et al. Novel mutations in ZP1: Expanding the mutational spectrum associated with empty follicle syndrome in infertile women. Clin Genet. 2021;99(4):583-7.

17. Yang P, Chen T, Liu Y, Hou Z, Wu K, Cao Y, et al. The critical role of ZP genes in female infertility characterized by empty follicle syndrome and oocyte degeneration. Fertil Steril. 2021;115(5):1259-69.

18. Huo M, Zhang Y, Shi S, Shi H, Liu Y, Zhang L, et al. Gene Spectrum and Clinical Traits of Nine Patients With Oocyte Maturation Arrest. Frontiers In Genetics. 2022;13:772143.

19. Zou T, Xi Q, Liu Z, Li Z, Hou M, Zhu L, et al. A Novel Homozygous Nonsense Mutation in ZP1 Causes Female Infertility due to Empty Follicle Syndrome. Reprod Sci. 2022.

20. Dai C, Hu L, Gong F, Tan Y, Cai S, Zhang S, et al. ZP2 pathogenic variants cause in vitro fertilization failure and female infertility. Genet Med. 2019;21(2):431-40.

21. Sun Y, Zeng Y, Chen H, Zhou Z, Fu J, Sang Q, et al. A novel homozygous variant in ZP2 causes abnormal zona pellucida formation and female infertility. J Assist Reprod Genet. 2021;38(5):1239-45.

22. Shen Y, Guo J, Zhang X, Wang X, Zhu S, Chen D, et al. Identification of a heterozygous variant of ZP2 as a novel cause of empty follicle syndrome in humans and mice. Human Reproduction (Oxford, England). 2022;37(4):859-72.

23. Jia W, Xi Q, Zhu L, Luo Y, Li Z, Hou M, et al. Novel mutations in ZP2 and ZP3 cause female infertility in three patients. J Assist Reprod Genet. 2022;39(5):1205-15.

24. Hou M, Zhu L, Jiang J, Liu Z, Li Z, Jia W, et al. Novel Heterozygous Mutations in ZP2 Cause Abnormal Zona Pellucida and Female Infertility. Reprod Sci. 2022.

25. Chen T, Bian Y, Liu X, Zhao S, Wu K, Yan L, et al. A Recurrent Missense Mutation in ZP3 Causes Empty Follicle Syndrome and Female Infertility. Am J Hum Genet. 2017;101(3):459-65.

26. Zhang D, Zhu L, Liu Z, Ren X, Yang X, Li D, et al. A novel mutation in ZP3 causes empty follicle syndrome and abnormal zona pellucida formation. J Assist Reprod Genet. 2021;38(1):251-9.

27. Chen Y, Wang Z, Wu Y, He W, Du J, Cai S, et al. Case Report: A Novel Heterozygous ZP3 Deletion Associated With Empty Follicle Syndrome and Abnormal Follicular Development. Frontiers in Genetics. 2021;12.

28. Zhang Z, Guo Q, Jia L, Zhou C, He S, Fang C, et al. A novel gene mutation in ZP3 loop region identified in patients with empty follicle syndrome. Hum Mutat. 2022;43(2):180-8.

29. Wei X, Li Y, Liu Q, Liu W, Yan X, Zhu X, et al. Mutations in ZP4 are associated with abnormal zona pellucida and female infertility. J Clin Pathol. 2022;75(3):201-4.

**literature included in the study**
